# Paternal and maternal exposures to adverse childhood experiences and spontaneous fetal loss: a nationwide cross-sectional analysis

**DOI:** 10.1186/s12889-024-18477-y

**Published:** 2024-04-15

**Authors:** Wen Liu, Weidi Sun, Lili Yang, Yizhou Huang, Siyu Zhu, Wenhan Xiao, Siqing Cheng, Jiajun Hao, Jiayao Ying, Hanlu Chen, Ziyang Ren, Shuhui Wang, Peige Song

**Affiliations:** 1 School of Public Health and the Second Affiliated Hospital, Zhejiang University School of Medicine, Hangzhou, Zhejiang China; 2 Department of Nursing, The Fourth Affiliated Hospital, International institutes of Medicine, Zhejiang University School of Medicine, Yiwu, Zhejiang China; 3 Women’s Hospital, Zhejiang University School of Medicine, Hangzhou, Zhejiang China; 4 International School of Medicine, Zhejiang University School of Medicine, Yiwu, Zhejiang China; 5 Center for Global Health, Zhejiang University School of Medicine, Hangzhou, Zhejiang China; 6 Institute of Reproductive and Child Health / Key Laboratory of Reproductive Health, National Health Commission of the People’s Republic of China, Peking University, Beijing, China; 7 Department of Epidemiology and Biostatistics, School of Public Health, Peking University, Beijing, China

**Keywords:** Adverse childhood experience, Intra-familial ACEs, Extra-familial ACEs, Spontaneous fetal loss

## Abstract

**Background:**

Adverse childhood experiences (ACEs) might be associated with maternal spontaneous fetal loss, while evidence among Chinese population is limited. This study aims to explore the associations of adverse childhood experiences (ACEs) among women and their spouses with the risk of spontaneous abortion and stillbirth.

**Method:**

Data were from the China Health and Retirement Longitudinal Study (CHARLS) 2014 survey. ACEs were categorized into intra-familial ACEs and extra-familial ACEs. The associations of maternal and paternal ACEs with women’s history of spontaneous abortion and stillbirth were investigated by logistic regression.

**Results:**

7,742 women were included with 9.05% and 2.47% experiencing at least one spontaneous abortion or stillbirth, respectively. Women exposed to 2, 3, and ≥ 4 ACEs were at significantly higher odds of spontaneous abortion, with adjusted odds ratios (ORs) of 1.52 (95% [CI, Confidence Interval] 1.10–2.10), 1.50 (95% CI 1.07–2.09) and 1.68 (95% CI 1.21–2.32), respectively. A significant association between ≥ 4 maternal intra-familial ACEs and stillbirth (OR 2.23, 95% CI 1.12–4.42) was also revealed. Furthermore, paternal exposures to 3 and ≥ 4 overall ACEs were significantly associated with their wives’ history of spontaneous abortion, with adjusted ORs of 1.81 (95% CI 1.01–3.26) and 1.83 (95% CI 1.03–3.25), respectively.

**Conclusion:**

Both maternal and paternal ACEs were associated with spontaneous abortion, and potential mediators might need to be considered to further explore impacts of maternal and paternal ACEs on maternal reproductive health.

**Supplementary Information:**

The online version contains supplementary material available at 10.1186/s12889-024-18477-y.

## Background

According to the United Nations Children’s Fund (UNICEF) and World Health Organization (WHO), spontaneous abortion and stillbirth are two primary reasons for spontaneous fetal loss, but have received little research attention [1, 2]. Spontaneous abortion is a loss of pregnancy before viability [1]. Recent reports suggested 10–14% of recognized pregnancies could end up with spontaneous abortion in China, which was aligned with the world average of 15.3% and there were an estimated 23 million spontaneous abortions worldwide each year [1, 3]. Similarly, stillbirth is another common cause of reproductive failure, referring to infants born without signs of life [4]. Though the stillbirth rate keeps reducing globally with 18.4 per 1,000 total births as estimated in 2015, fetal death was still prevalent in China. For instance, an above global average stillbirth rate (35.5 per 1000 total births) was reported from six Chinese tertiary hospitals in 2015 and if the current trend is maintained, there will be 43,700 stillbirths in China in 2030 [4–6]. Women who have experienced spontaneous abortion and stillbirth can suffer from physical and psychological multimorbidity, such as obstetric complications, cardiovascular diseases, and depression, in which their spouses could also experience long-term adverse health outcomes [1, 4, 7]. In addition, due to the stigma associated with women who spontaneously lose a fetus, healthcare professionals and even the whole society are becoming more concerned about the undervalued economic implications of spontaneous abortion and stillbirth [5, 8, 9].

Although the high burden to individuals and society has called for population-level action to prevent spontaneous fetal loss, this will not be sufficient for people living with adverse childhood experiences (ACEs) [8, 10]. ACEs refer to a wide spectrum of intra- and extra-familial traumatic events to which children and adolescents may be exposed, such as abuse, neglect and household dysfunction [11–14]. In China, recent research demonstrated that an extremely large number of Chinese elderly have experienced at least 1 ACE during their lifetime, with 80% of 11,972 recorded [14]. From the intergenerational perspective, previous studies from England and America have revealed that women living with ACEs have been at a considerable disadvantage with respect to poor maternal and birth outcomes [15, 16]. A biological model suggests ACEs might accumulate negative emotions and inflammation in the immune system, subsequently relating to a limited capacity of fetal viability in utero [17]. Consistent with it, an emerging body of literature has demonstrated the associations between certain types of maternal ACEs and women’s reproductive outcomes, with particular examples of sexual abuse [18]. However, due to variations in cultural or ethnic differences in emotion regulation, the consequences of ACEs on spontaneous fetal loss in Chinese women may differ from those in English and American women [15, 16]. Furthermore, few studies have explored whether the developing fetus is susceptible to paternal ACEs. The absence of the father’s role in offspring upbringing has led previous explorations to overlook the impact of the fathers’ circumstances, such as ACEs, on maternal and child health [19]. Fathers may affect fetal outcomes through more overt means, such as aggressive behavior, substance abuse, and intimate relationship violence, rather than by disclosing owing to masculinity norms, which increases the risk of fetal death [20, 21].

Therefore, we hypothesized that maternal and paternal ACEs may be associated with an increased risk of spontaneous abortion and stillbirth in Chinese women. To address this research gap, this study retrospectively explored such associations based on the China Health and Retirement Longitudinal Study (CHARLS).

## Methods

### Study population

**Fig. 1:**
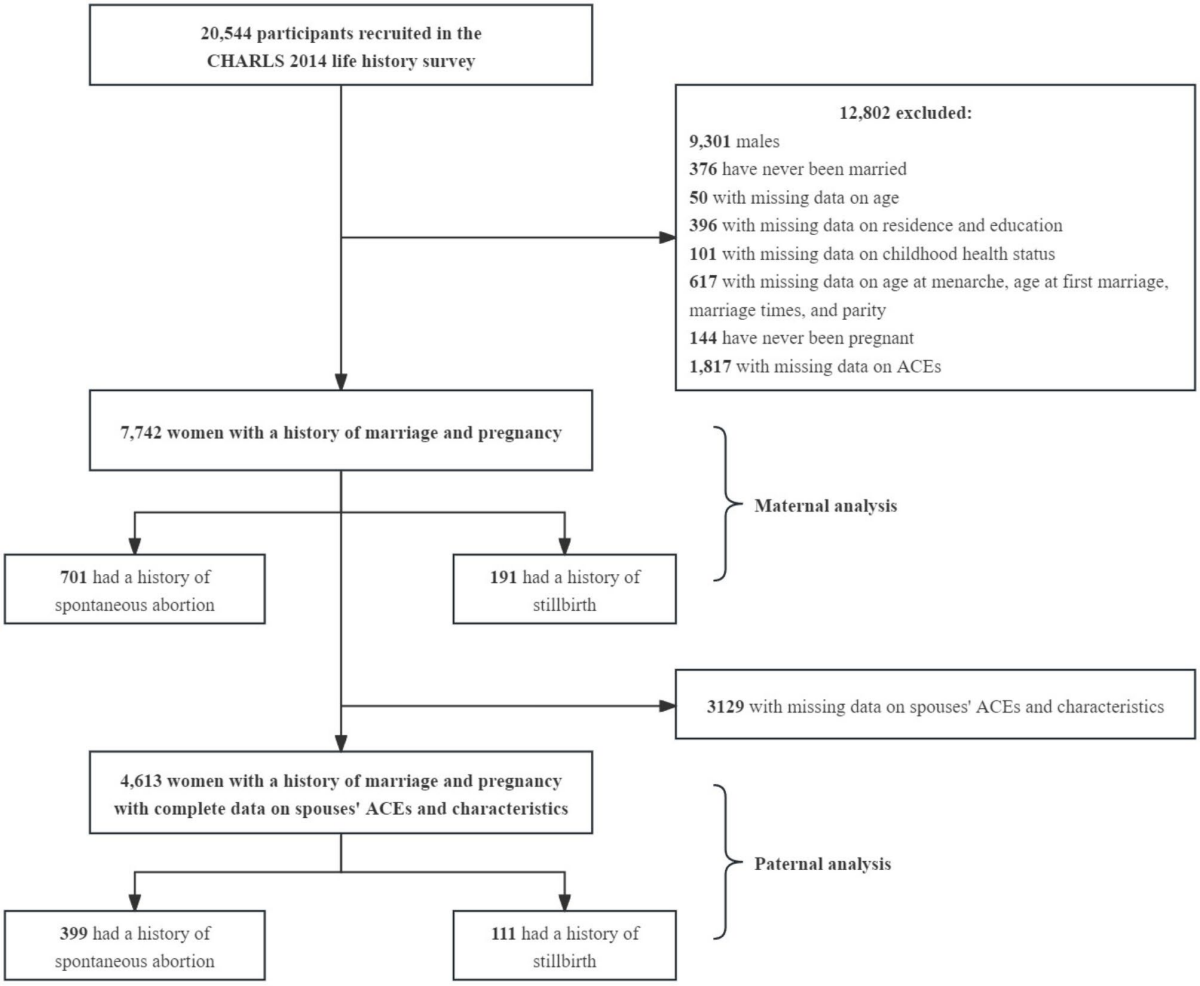
Flow chart of study participants selection. *Note* CHARLS, China Health and Retirement Longitudinal Study. ACEs, adverse childhood experiences

Data were from the 2014 life history survey of the CHARLS, a national cohort that collected extensive information on adults aged 45 or older in 28 provinces across China [22]. The CHARLS has been conducted since 2011 in which multistage sampling was utilized generally with Probability proportional to size (PPS) at the county/district and community level. A total of 20,544 participants were recruited to complete the life history survey through one-on-one interviews by trained field workers in 2014. Those who were males (*n* = 9,301), have never been married (*n* = 376), with missing data on age (*n* = 50), on residence and education (*n* = 396), on health status in childhood (*n* = 101), on age at menarche, age at the first marriage, marriage times, and parity (*n* = 617), have never been pregnant (*n* = 144), and had missing data on ACEs (*n* = 1,817) were excluded, leaving 7,742 participants for analysis of associations between maternal ACEs and women’s fetal loss. In the second part of association between paternal ACEs and fetal loss, women without data on their spouses’ ACEs and characteristics (*n* = 3,129) were further excluded. Inclusion and exclusion criteria for participants are as presented in the flow chart (Fig. 1).The CHARLS was approved by the Ethical Review Committee of the Peking University and implemented by the China Centre for Economic Research of Peking University (Number: IRB00001052-11014 and IRB00001052-11015). Informed written consent was obtained from all participants.

### Definition of ACEs

Responses to these ACE domains were self-reported by participants. Table S1 shows the detailed questions and definitions of ACEs [14]. Amongst, intra-familial ACEs involved 11 types, namely physical abuse, emotional neglect, economic adversity, family violence, parental separation or divorce, parental substance abuse, parents incarcerated, parental mental illness, parental disability, parental death, and sibling death, while extra-familial ACEs included 3 types, which were bullying, loneliness and community violence. The number of experienced types of ACEs was used to represent the extent of individuals’ adverse experiences, in which overall ACEs and intra-familial ACEs were further classified into five levels: 0, 1, 2, 3, and ≥ 4, while extra-familial ACEs were classified into 3 levels: 0, 1, and ≥ 2 [12].

### Assessment of spontaneous abortion and stillbirth

Self-reported pregnancy history was collected through face-to-face interviews. Spontaneous fetal loss including spontaneous abortion and stillbirth as the primary outcomes was assessed through questions: ‘Did you have induced abortion, spontaneous abortion, or stillbirth before the first biological child was born/during the period between the n^th^ biological child and the (*n* + 1)^th^ biological child was born/after the last biological child was born’ and ‘Did this pregnancy end up with induced abortion, miscarriage or stillbirth?’. Hence, a history of spontaneous abortion or stillbirth was defined once the participants answered ‘miscarriage’ or ‘stillbirth’. The total number of spontaneous abortions or stillbirths was the sum of the number of ‘miscarriages’ or ‘stillbirths’ that participants answered.

### Covariates’ assessment

The residence was divided into urban and rural. The highest education completed was classified as primary school or less, middle school, and high school or higher. Maternal age was derived by subtracting women’s birth year from 2014 and treated as a continuous variable. Age at menarche was categorized into < 16, 16–18 years, or > 18 years according to categories undertaken in CHARLS. Age at the first marriage was self-reported and treated as a continuous variable. Health status in childhood was measured by participants’ self-reported health condition compared to their peers before the age of 15. Those reporting “much healthier”, “somewhat healthier”, or “about average” were classified as healthy, and those responding “somewhat less healthy” or “much less healthy” were classified as unhealthy [22]. Since most women only married once, marriage times were defined as 1 and more than 1. Parity was also binarized as less than 3, and 3 or more.

### Statistical analysis

The baseline characteristics were described as medians and interquartile ranges (IQRs) for continuous variables, and frequency and percent (%) for categorical variables. To compare the characteristics of included participants by the number of ACEs, differences in continuous variables and categorical variables across ACE groups were assessed by Wilcoxon rank sum tests and Chi-square tests. The logistic regression was first conducted to investigate the associations of maternal overall ACEs, intra-familial ACEs, extra-familial ACEs and each type of maternal ACEs with the maternal history of spontaneous abortion and stillbirth, respectively. Linear regression was performed to assess the association between maternal ACEs and counts of spontaneous abortion and stillbirth. All odds ratios (ORs), β, and 95% confidence intervals (CIs) were adjusted for maternal age, maternal residence, maternal education, maternal age at the first marriage, age at menarche, maternal health status in childhood, parity, and maternal marriage times. *P* values for trends were undertaken as well.

The logistic regression was then conducted to investigate the associations of paternal overall ACEs, intra-familial ACEs, extra-familial ACEs and each type of paternal ACEs with the history of spontaneous abortion and stillbirth. All OR values were adjusted for maternal ACEs, maternal age, maternal residence, maternal education, maternal age at the first marriage, age at menarche, maternal health status in childhood, parity, and maternal marriage times, as well as paternal age, paternal education, paternal age at the first marriage, paternal health status in childhood, and paternal marriage times.

This study was reported according to Strengthening the Reporting of Observational Studies in Epidemiology (STROBE) guideline [23]. To clarify our study hypotheses, a directed acyclic graph of the analysis is presented in Figure S1. All statistical analyses were performed using SAS statistical software (version 9.4; SAS Institute Inc., Cary, NC, USA). All analyses were two-sided, and a *P* value of < 0.05 or a 95% CI that did not include 1.00 was considered statistically significant.

## Results

7,742 women with a history of marriage and pregnancy were included in the maternal analysis and the baseline characteristics of women are shown in Table 1. Amongst, 701 had a history of spontaneous abortion and 191 had a history of stillbirth. We also found that 91.50% of those in our study have experienced at least 1 ACE; 9.05% and 2.47% have experienced at least one spontaneous abortion and stillbirth, respectively. Generally, women who had ACEs were more likely to have a rural residence, lower education attainment, worse health status in childhood, earlier age at the first marriage, more marriage times, more parity, and more total number of spontaneous abortions, compared to those who did not have ACEs (all *P* values < 0.05).

**Table 1:** Baseline characteristics of women with a history of marriage and pregnancy by number of ACEs

		No. of ACEs (N = 7,742)					*P* value
		0 (N = 658)	1 (N = 1,512)	2 (N = 1,789)	3 (N = 1,462)	4 or more (N = 2,321)	
Age, years		55.0 (50.0–63.0)	56.0 (49.0–64.0)	56.0 (49.0–63.0)	56.0 (49.0–63.0)	56.0 (48.0–64.0)	0.627
Residence							< 0.001
	Rural	360 (54.7)	816 (54.0)	983 (55.0)	892 (61.0)	1406 (60.6)	
	Urban	298 (45.3)	696 (46.0)	806 (45.1)	570 (39.0)	915 (39.4)	
Maternal highest education completed							< 0.001
	Primary school or less	389 (59.1)	926 (61.2)	1131 (63.2)	972 (66.5)	1661 (71.6)	
	Middle school	166 (25.2)	389 (25.7)	417 (23.3)	334 (22.9)	480 (20.7)	
	High school or higher	103 (15.7)	197 (13.0)	241 (13.5)	156 (10.7)	180 (7.8)	
Maternal age at menarche, years							0.607
	< 16	296 (45.0)	683 (45.2)	807 (45.1)	638 (43.6)	994 (42.8)	
	16–18	267 (40.6)	629 (41.6)	747 (41.8)	621 (42.5)	1030 (44.4)	
	> 18	95 (14.4)	200 (13.2)	235 (13.1)	203 (13.9)	297 (12.8)	
Maternal health status in childhood							< 0.001
	Healthy	627 (95.3)	1383 (91.5)	1612 (90.1)	1287 (88.0)	1841 (79.3)	
	Unhealthy	31 (4.7)	129 (8.5)	177 (9.9)	175 (12.0)	480 (20.7)	
Maternal age at the first marriage, years		22.0 (20.0–24.0)	22.0 (20.0–24.0)	22.0 (20.0–24.0)	21.0 (20.0–24.0)	21.0 (19.0–23.0)	< 0.001
Maternal marriage times							0.021
	1	639 (97.1)	1462 (96.7)	1737 (97.1)	1395 (95.4)	2218 (95.6)	
	1 or more	19 (2.9)	50 (3.3)	52 (2.9)	67 (4.6)	103 (4.4)	
Parity							0.011
	0	41 (6.2)	68 (4.5)	82 (4.6)	65 (4.5)	108 (4.7)	
	1	120 (18.2)	287 (19.0)	363 (20.3)	261 (17.9)	393 (16.9)	
	2	263 (40.0)	562 (37.2)	606 (33.9)	528 (36.1)	811 (34.9)	
	3 or more	234 (35.6)	595 (39.4)	738 (41.3)	608 (41.6)	1009 (43.5)	
History of spontaneous abortion							< 0.001
	No	613 (93.2)	1403 (92.8)	1632 (91.2)	1328 (90.8)	2065 (89.0)	
	Yes	45 (6.8)	109 (7.2)	157 (8.8)	134 (9.2)	256 (11.0)	
History of stillbirth							0.195
	No	650 (98.8)	1478 (97.8)	1743 (97.4)	1425 (97.5)	2255 (97.2)	
	Yes	8 (1.2)	34 (2.3)	46 (2.6)	37 (2.5)	66 (2.8)	

*Note* ACEs, adverse childhood experiences. Continuous variables including maternal age, age at menarche, maternal age at the first marriage were compared using Wilcoxon rank sum test and are presented as medians (M) with interquartile ranges (IQRs). Categorical variables including maternal residence, maternal highest education completed, maternal health status in childhood, maternal marriage times, parity, history of spontaneous abortion and history of stillbirth were compared using Chi-square test and are presented as number (N) with percent (%)

### Association of maternal ACEs with spontaneous abortion and stillbirth

As shown in Table 2, women who had ≥ 4 overall ACEs and ≥ 2 extra-familial ACEs were significantly associated with a history of spontaneous abortion, based on the adjusted ORs of 1.66 (95% CI 1.19–2.31) for 4 or more overall ACEs and 1.48 (95% CI 1.14–1.93) for ≥ 2 extra-familial ACEs, respectively. We also found that women who experienced 2, 3, and ≥ 4 intra-familial ACEs were at significantly higher risk of spontaneous abortion, with adjusted ORs of 1.52 (95% CI 1.10–2.10), 1.50 (95% CI 1.07–2.09) and 1.68 (95% CI 1.21–2.32), respectively. However, we only found a significant association between ≥ 4 intra-familial ACEs and a history of stillbirth (OR 2.23, 95% CI 1.12–4.42). Similar results were found for the association between maternal ACEs and counts of spontaneous abortion and stillbirth.

**Table 2:** Association of maternal ACEs with risks and counts of spontaneous abortion and stillbirth

		Risk, aOR (95% CI)		Count, *β* (95% CI)	
		Spontaneous abortion	Stillbirth	Spontaneous abortion	Stillbirth
**No. of maternal ACEs**	**N (%) of ACE categories**				
**Overall**					
0	658 (8.5)	Reference	Reference	Reference	Reference
1	1,512 (19.5)	1.04 (0.73–1.50)	1.78 (0.82–3.87)	0.007 (-0.026–0.040)	0.010 (-0.008–0.027)
2	1,789 (23.1)	1.29 (0.92–1.83)	2.01 (0.94–4.29)	0.022 (-0.010–0.055)	0.012 (-0.006–0.029)
3	1,462 (18.9)	1.37 (0.96–1.95)	1.91 (0.88–4.14)	0.027 (-0.006–0.060)	0.009 (-0.008–0.027)
4 or more	2,321 (30.0)	1.66 (1.19–2.31)**	1.97 (0.93–4.15)	0.052 (0.200–0.083)**	0.012 (-0.005–0.029)
*P* for trend		< 0.001	0.265	< 0.001	0.17
**Intra-familial**					
0	778 (10.1)	Reference	Reference	Reference	Reference
1	1,791 (23.1)	1.14 (0.81–1.59)	1.88 (0.94–3.76)	0.014 (-0.017–0.044)	0.012 (-0.005–0.028)
2	2,007 (25.9)	1.52 (1.10–2.10)*	1.70 (0.85–3.40)	0.035 (0.005–0.065)*	0.009 (-0.007–0.025)
3	1,470 (19.0)	1.50 (1.07–2.09)*	1.68 (0.82–3.42)	0.041 (0.010–0.072)*	0.007 (-0.010–0.024)
4 or more	1,696 (21.9)	1.68 (1.21–2.32)**	2.23 (1.12–4.42)*	0.052 (0.021–0.083)***	0.018 (0.002–0.035)*
*P* for trend		< 0.001	0.075	< 0.001	0.048
**Extra-familial**					
0	5,373 (69.4)	Reference	Reference	Reference	Reference
1	1,769 (22.8)	1.10 (0.91–1.33)	0.82 (0.57–1.19)	0.004 (-0.015–0.024)	-0.007 (-0.018–0.003)
2 or more	600 (7.8)	1.48 (1.14–1.93)**	1.03 (0.62–1.71)	0.049 (0.018–0.079)**	0.000 (-0.017–0.016)
*P* for trend		0.007	0.657	0.002	0.409

*Note* ACEs, adverse childhood experiences. aOR, adjusted odds ratio. CI, confidence interval. All OR and *β* values were adjusted for maternal age, maternal residence, maternal education, maternal age at the first marriage, age at menarche, maternal health status in childhood, parity, and maternal marriage times."*" means a *P* value <0.05. "**" means a *P* value <0.01. "***" means a *P* value <0.001.

With regard to each type of maternal ACE, we found that family violence, parental separation or divorce, parental substance abuse, sibling death, physical abuse, and bullying were significantly associated with the history of spontaneous abortion, with adjusted ORs of 1.30 (95% CI 1.10–1.54), 2.70 (95% CI 1.32–5.53), 1.25 (95% CI 1.07–1.46), 1.68 (95% CI 1.22–2.31), 1.19 (95% CI 1.01–1.42), and 1.44 (95% CI 1.19–1.74), respectively. However, none of these 14 maternal ACEs was significantly associated with stillbirth (Table S2).

### Association of paternal ACEs with spontaneous abortion and stillbirth

After excluding women with missing data on spousal ACEs and characteristics (*N* = 3,129), 4,613 pairs of women and their spouses were included in the analyses (Fig. 1). Amongst, 399 women had a history of spontaneous abortion, and 111 had a history of stillbirth. More details on baseline characteristics of women with available data on paternal ACEs were presented in Table S3. As shown in Table 3, the histories of 3 and ≥ 4 paternal overall ACEs were significantly associated with their counterpart’s history of spontaneous abortion, based on the adjusted ORs of 1.81 (95% CI 1.01–3.26) and 1.83 (95% CI 1.03–3.25), respectively. Also, the presence of a history involving 4 or more paternal intra-familial ACEs exhibited a statistically significant correlation with the risk of spontaneous abortion, as indicated by the adjusted OR of 1.80 (95% CI 1.08-3.00). However, no significant association was found between the number of paternal ACEs and their wives’ history of stillbirth. Regarding each type of paternal ACEs, we found that childhood experience of family violence and bullying were significantly associated with their wives’ history of spontaneous abortion, with adjusted ORs of 1.48 (95% CI 1.18–1.85) and 1.31 (95% CI 1.03–1.68), respectively (Table S4), while amongst paternal ACEs, family violence showed a lower risk with their wives’ history of stillbirth, with adjusted OR of 0.58 (95% CI 0.35–0.96).

**Table 3:** Association of paternal ACEs with risks of spontaneous abortion and stillbirth

	No. of ACEs					
	0	1	2 (2 or more)	3	4 or more	P for trend
***Spontaneous abortion***						
Paternal overall	Reference	1.66 (0.91–3.01)	1.77 (0.99–3.16)	1.81 (1.01–3.26)*	1.83 (1.03–3.25)**	0.171
Paternal intra-familial	Reference	1.67 (0.99–2.79)	1.40 (0.84–2.34)	1.58 (0.94–2.66)	1.80 (1.08–3.00)**	0.086
Paternal extra-familial	Reference	1.16 (0.91–1.48)	1.19 (0.81–1.75)			0.189
***Stillbirth***						
Paternal overall	Reference	1.16 (0.42–3.17)	1.06 (0.40–2.85)	1.37 (0.51–3.66)	1.27 (0.49–3.30)	0.527
Paternal intra-familial	Reference	1.74 (0.66–4.61)	1.44 (0.55–3.79)	1.67 (0.63–4.43)	1.31 (0.49–3.50)	0.841
Paternal extra-familial	Reference	1.24 (0.80–1.91)	0.93 (0.44–1.98)			0.715

*Note* ACEs, adverse childhood experiences. OR, odds ratio. CI, confidence interval. All OR values were adjusted for maternal ACEs, maternal age, maternal residence, maternal education, maternal age at the first marriage, age at menarche, maternal health status in childhood, parity, and maternal marriage times, as well as paternal age, paternal education, paternal age at the first marriage, paternal health status in childhood, and paternal marriage times. Item “2” in the “No. of ACEs” section is for overall and intra-familial ACEs while item “2 or more” is for extra-familial ACEs. "*" means a *P* value<0.05. "**" means a *P* value <0.01. "***" means a *P* value <0.001.

## Discussion

Using a Chinese population-based sample of aged population, we demonstrated that both maternal and paternal intra-familial ACEs were associated with spontaneous abortion. Moreover, results from our study indicated that a large majority of Chinese elderly (above 90%) experienced at least 1 ACE in the past and it is higher than similar previous research in China and other countries [14, 24]. In addition, compared to previous reports on the incidence of spontaneous abortion and stillbirth in China, our study showed similar results with around 9% and 3%, respectively, which are also close to the global averages [3, 4]. This nearly unchanged fluctuation assures that urgent and effective interventions to prevent such adverse reproductive events in Chinese women are still entailed.

Associations between elevated levels of maternal ACEs and adverse pregnancy outcomes were frequently reported around the globe previously. A recent cohort study in England by Demakakos, et al. [24] reassures our findings that women with ≥ 3 ACEs were more likely to experience a single spontaneous abortion compared with women with no ACEs. Moreover, a higher risk of experiencing a stillbirth among women who had 1, 3, or more ACEs is worth being concerned about in our study. Previous research from Wisconsin, United States in 2019 has also reported that an increased risk of spontaneous abortion and stillbirth is associated with each additional ACE among low-income women [25]. Our study also found evidence indicating that maternal exposure to ACEs within the family was significantly correlated with higher rates of spontaneous abortion and stillbirth. This could be explained by that exposure to ACEs beyond the family may accelerate the early onset of risky health behaviours, such as smoking and drinking [26]. Consequently, these behaviours might contribute to poor maternal health prior to pregnancy, ultimately leading to adverse outcomes during gestation for women [27–29].

A few hypotheses through socioeconomic, biological, psychological, and behavioural pathways have been provided to understand the causal mechanisms of maternal ACEs leading to spontaneous fetal loss [24, 28]. For example, childhood psychological adversity which is associated with cumulative and chronic exposure to stress might contribute to broad alterations in an individual’s neural, endocrine, immune, and metabolic systems [30, 31]. Any of those changes, such as neuroendocrine disruption and immunologic dysregulation, could then have an impact on the vital pregnancy processes and women’s reproductive environment during early pregnancy, contributing to spontaneous abortion and stillbirth [24, 28]. Additionally, hormonal factors such as early age at menarche and menopause have been related to reproductive dysfunctions including spontaneous fetal loss but also have been associated with poor-quality parenting as ACEs reflect [11, 21]. Moreover, experiencing ACEs has also been linked to higher rates of substance abuse, sexually transmitted infections (STIs), and risky sexual behaviours, which have been identified as risk factors for fetal death [28, 32].

Previous studies on the association between specific type of maternal ACEs and fetal loss were often inconsistent. In our study, family violence, parental separation or divorce, parental substance abuse, sibling death, physical abuse, and bullying were associated with maternal risk of experiencing spontaneous abortion. Most types of ACEs were largely reported in terms of their adverse impact on maternal reproductive health [28, 29]. However, maternal emotional neglect is usually identified to be associated with fetal loss [29, 33] while not evident in our study and the association of each maternal ACE was not observed for stillbirth as well. For example, a case-control study in the U.S. indicated that there was no significant association between child maltreatment and risk of stillbirth except for emotional neglect [33]. While according to an Australian study, higher rates of spontaneous abortion were persistently associated with maternal exposure to emotional neglect, but also linked to childhood maltreatment, even though slightly attenuated after adjustment [32]. Furthermore, the association between substance abuse and spontaneous abortion was also reported previously, however, inconsistent measurements are worth concerning. In the study by Li, et al., the substance abuse measurement for childhood included the item “you had a problem because of alcohol or drugs”, indicating that in their analysis, women were not only exposed to problematic alcohol or drug use from others but also themselves, which was not covered in our study [17]. For another, the substances measured in our study ranged from alcohol, smoking, drugs, and gambling while in Li, et al. study, only alcohol and drugs were included [17]. Therefore, further investigation with comparable substances in different populations is warranted.

A paucity of research has explored the association of paternal ACEs with their wives’ history of stillbirth and spontaneous abortion. Previous evidence showed ACEs were positively associated with adult family health including unhealthy marriage relationships [34], supporting our findings regarding the deleterious impact of men’s ACEs on their wives’ reproductive health. However, this finding should be interpreted carefully due to the impact of potential mediators such as maternal health conditions and paternal behaviours. For example, an increased risk of intimate partner violence perpetration among adult men with ACEs was recorded which might exert a higher risk on their wives’ physical and mental health, resulting in potential fetal loss [35]. In addition to this, our findings suggested the differentiated impacts of ACEs on spontaneous abortion and stillbirth and such differences might be driven by various risk factors of spontaneous abortion and stillbirth. For example, evidence showed that spontaneous abortion could be much more sensitive than stillbirth to subtle emotional, behavioural and environmental changes, including the impact from spouses [1, 4].

An advantage of our study is the large sample size (> 5000) in China with relatively detailed and comparable spontaneous fetal loss outcomes and socioeconomic data compared to previous research [25, 33]. Additionally, our study analyzed spontaneous abortion and stillbirth as independent outcomes, thus providing more explicit differences in associations between maternal and paternal ACEs and women’s reproductive events.

There were several limitations in this study. First, recall bias might be worth noting in our study since we used the existing dataset and the cross-sectional survey conducted in 2014 [36] that relied on retrospective reports. Second, a limited number of spontaneous abortions and stillbirths were reported in our study. For participants answering questions through face-to-face interviews, confidential data on ACEs, stillbirth, and spontaneous abortion could be also under-reported due to the sensitivity of the topics, and stigma and discrimination around the topic [37, 38]. Third, the absence of data concerning potential confounding factors that could significantly influence pregnancy loss - such as marital relationships, the status of smoking and alcohol use during pregnancy - hindered our ability to account for their impact. Further investigation is warranted to comprehensively address these factors in subsequent research endeavors. Additionally, given the characteristics of the cross-sectional study [39], we could not provide any causal relationship between experiencing ACEs and spontaneous abortion and stillbirth.

## Conclusion

Women who experienced ACEs and had spouses with ACEs were more likely to develop spontaneous abortion or stillbirth. Acknowledging and identifying the impact of maternal and paternal ACEs on women, especially their maternal health and wellbeing, will allow for further prevention and management of pregnancies and the development of offspring.

### Electronic supplementary material

Below is the link to the electronic supplementary material.

Supplementary Material 1

## Data Availability

The data that support the findings of this study are available from the website of China Health and Retirement Longitudinal Study at http://charls.pku.edu.cn/en.

## Abbreviations

ACEs: Adverse Childhood Experiences
CHARLS: China Health and Retirement Longitudinal Study
UNICEF: United Nations Children’s Fund (UNICEF)
WHO: World Health Organization
PPS: Probability proportional to size
IQR: interquartile range
