# Supplementary material for: Paternal and maternal exposures to adverse childhood experiences and spontaneous fetal loss: a nationwide cross-sectional analysis
Source: BMC Public Health. 2024 Apr 15;24:1047. doi: 10.1186/s12889-024-18477-y (PMC11020413; doi:10.1186/s12889-024-18477-y)
Supplement: Supplementary file 1 — Supplementary Material 1 [file 12889_2024_18477_MOESM1_ESM.docx]

**Table and Figure Legends**

**Table S1. Definitions of ACEs in the CHARLS**

Note: ACEs, adverse childhood experiences. CHARLS, China Health and Retirement Longitudinal Study.

**Table S2. Association between each type of maternal ACEs and history of spontaneous abortion and stillbirth**

Note: ACEs, adverse childhood experiences. OR, odds ratio. CI, confidence interval. All OR values were adjusted for maternal age, maternal residence, maternal education, maternal age at the first marriage, age at menarche, maternal health status in childhood, parity, and maternal marriage times. Bolded means statistically significant.

**Table S3. Baseline characteristics of women with a history of marriage and pregnancy and with complete data on paternal ACEs**

Note: ACEs, adverse childhood experiences. Continuous variables are presented as medians (M) with interquartile ranges (IQRs), and categorical variables are presented as numbers (N) with percent (%).

**Table S4. Association between each type of paternal ACEs and history of spontaneous abortion and stillbirth**

Note: ACEs, adverse childhood experiences. OR, odds ratio. CI, confidence interval. All OR values were adjusted for maternal ACEs, maternal age, maternal residence, maternal education, maternal age at the first marriage, age at menarche, maternal health status in childhood, parity, and maternal marriage times, as well as paternal age, paternal education, paternal age at the first marriage, paternal health status in childhood, and paternal marriage times. Bolded means statistically significant. “-” refers to insufficient statistical power.

**Figure S1. Directed Acyclic Graph of the study hypothesis**

Note: ACEs, adverse childhood experiences.

Table S1. Definition of ACEs in the CHARLS

| **Types of ACEs** | | **Questionnaire Items** | **Answers defined as exposure to each type** |
| --- | --- | --- | --- |
| Intra-familial | Emotional neglect | How much love and affection did your female guardian give you while you were growing up? | rarely/never |
|  |  | How much effort did your female guardian put into watching over you? | a little/not at all |
|  |  | Did your male/female guardian treat your siblings better than you when you were growing up? | very strict/somewhat strict |
|  | Family violence | Did your parents often quarrel? | often/sometimes |
|  |  | Have your father/mother ever beat up your mother/father? | often/sometimes |
|  | Parental separation or divorce | Were your biological parents divorced (including long separation due to emotional problems) before you were 17 years? | yes |
|  | Parental substance abuse | During the years you were growing up, which one of the followings did your male/female guardian ever have? | alcoholism/smoking/drug/gambling |
|  | Parents incarcerated | During the years you were growing up, which one of the followings did your male/female guardian ever have? | arrested/sent to prison |
|  | Parental mental illness | During the years you were growing up, had your male/female guardian showed continued signs of sadness or depression that lasted two weeks or more? | yes |
|  |  | Was this problem of your male/female guardian, sadness or depression during all, most, some, or only a little of your childhood? | all/most |
|  |  | Did your male/female guardian have abnormality of mind when you were young? | yes |
|  | Parental disability | Did your male/female guardian have a long time be sick on bed when you were young? | yes |
|  |  | Did your male/female guardian have a serious deformity when you were young? | yes |
|  | Parental death | Either of the parents was dead before participant was 17 years. | yes |
|  | Sibling death | Any of the siblings was dead before participant was 17 years. | yes |
|  | Physical abuse | When you were growing up, did your male/female guardian ever hit you? | often/sometimes |
|  |  | When you were growing up, how often did your brother or sister ever hit you? | often/sometimes |
|  | Economic adversity | When you were a child before age 17, compared to the average family in the same community/village at that time, how was your family’s financial situation? | a lot/somewhat worse off than them |
| Extra-familial | Bullying | When you were a child, how often were you picked on or bullied by kids in your neighborhood? | often/sometimes |
|  |  | When you were a child, how often were you picked on or bullied by kids in your school? | often/sometimes |
|  |  | When you were a child, how often did you feel worried about your physical safety at school? | often/sometimes |
|  | Loneliness | When you were a child, how often did you feel lonely for not having friends? | often/sometimes |
|  | Community violence | Was it safe being out alone at night in the neighborhood where you lived as a child? | not very safe/not safe at all |
|  |  | Were the neighbors of the place where you lived as a child very close-knit? | not very close-knit/not close-knit at all |

Note: ACEs, adverse childhood experiences. CHARLS, China Health and Retirement Longitudinal Study.

Table S2. Association between each type of maternal ACEs and history of spontaneous abortion and stillbirth

| **Types of ACEs** | **Spontaneous abortion** | **Stillbirth** |
| --- | --- | --- |
|  | **OR (95% CI)** | |
| ***Intra-familial*** | | |
| Emotional neglect | 0.96 (0.82 to 1.12) | 1.01 (0.76 to 1.36) |
| Family violence | **1.30 (1.10 to 1.54)** | 0.89 (0.63 to 1.25) |
| Parental separation or divorce | **2.70 (1.32 to 5.53)** | 0.75 (0.10 to 5.49) |
| Parental substance abuse | **1.25 (1.07 to 1.46)** | 1.20 (0.90 to 1.62) |
| Parents incarcerated | 0.64 (0.08 to 4.92) | 2.09 (0.27 to 16.39) |
| Parental mental illness | 1.19 (0.95 to 1.48) | 1.24 (0.85 to 1.82) |
| Parental disability | 1.08 (0.90 to 1.31) | 1.34 (0.97 to 1.86) |
| Parental death | 0.86 (0.67 to 1.12) | 1.31 (0.88 to 1.95) |
| Sibling death | **1.68 (1.22 to 2.31)** | 1.25 (0.69 to 2.28) |
| Physical abuse | **1.19 (1.01 to 1.42)** | 1.04 (0.75 to 1.44) |
| Economic adversity | 1.13 (0.96 to 1.33) | 1.21 (0.90 to 1.64) |
| ***Extra-familial*** | | |
| Bullying | **1.44 (1.19 to 1.74)** | 1.00 (0.67 to 1.48) |
| Loneliness | 1.07 (0.83 to 1.36) | 1.09 (0.71 to 1.67) |
| Community violence | 1.16 (0.92 to 1.46) | 0.73 (0.45 to 1.17) |

Note: ACEs, adverse childhood experiences. OR, odds ratio. CI, confidence interval. All OR values were adjusted for maternal age, maternal residence, maternal education, maternal age at the first marriage, age at menarche, maternal health status in childhood, parity, and maternal marriage times. Bolded means statistically significant.

Table S3. Baseline characteristics of women with a history of both marriage and pregnancy and with complete data on paternal ACEs

| **Characteristics** | | **No. of ACEs (N=4,613)** | | | | | ***P* value** |
| --- | --- | --- | --- | --- | --- | --- | --- |
|  |  | **0 (N=425)** | **1 (N=939)** | **2 (N=1,046)** | **3 (N=881)** | **4 or more (N=1,322)** |  |
| Age, years | | 54.0 (49.0-62.0) | 55.0 (48.0-63.0) | 55.0 (48.0-62.0) | 54.0 (48.0-61.0) | 53.0 (48.0-61.0) | 0.263 |
| Paternal age, years | | 56.0 (50.0-63.0) | 57.0 (50.0-64.0) | 57.0 (50.0-64.0) | 57.0 (49.0-64.0) | 56.0 (49.0-64.0) | 0.747 |
| Residence | | | |  |  |  | 0.003 |
|  | Rural | 257 (60.5) | 534 (56.9) | 603 (57.7) | 566 (64.3) | 827 (62.6) |  |
|  | Urban | 168 (39.5) | 405 (43.1) | 443 (42.4) | 315 (35.8) | 495 (37.4) |  |
| Maternal highest education completed | | | |  |  |  | <0.001 |
|  | Primary school or less | 244 (57.4) | 564 (60.1) | 645 (61.7) | 582 (66.1) | 917 (69.4) |  |
|  | Middle school | 123 (28.9) | 255 (27.2) | 260 (24.9) | 212 (24.1) | 296 (22.4) |  |
|  | High school or higher | 58 (13.7) | 120 (12.8) | 141 (13.5) | 87 (9.9) | 109 (8.3) |  |
| Paternal highest education completed | | | |  |  |  | <0.001 |
|  | Primary school or less | 145 (34.1) | 371 (39.5) | 425 (40.6) | 377 (42.8) | 606 (45.8) |  |
|  | Middle school | 172 (40.5) | 369 (39.3) | 386 (36.9) | 316 (35.9) | 478 (36.2) |  |
|  | High school or higher | 108 (25.4) | 199 (21.2) | 235 (22.5) | 188 (21.3) | 238 (18.0) |  |
| Age at menarche, years | |  |  |  |  |  | 0.350 |
|  | <16 | 198 (46.6) | 428 (45.6) | 466 (44.6) | 396 (45.0) | 576 (43.6) |  |
|  | 16-18 | 166 (39.1) | 404 (43.0) | 444 (42.5) | 371 (42.1) | 600 (45.4) |  |
|  | >18 | 61 (14.4) | 107 (11.4) | 136 (13.0) | 114 (12.9) | 146 (11.0) |  |
| Maternal health status in childhood | |  |  |  |  |  | <0.001 |
|  | Healthy | 408 (96.0) | 860 (91.6) | 937 (89.6) | 785 (89.1) | 1066 (80.6) |  |
|  | Unhealthy | 17 (4.0) | 79 (8.4) | 109 (10.4) | 96 (10.9) | 256 (19.4) |  |
| Paternal health status in childhood | |  |  |  |  |  | <0.001 |
|  | Healthy | 390 (91.8) | 861 (91.7) | 950 (90.8) | 776 (88.1) | 1138 (86.1) |  |
|  | Unhealthy | 35 (8.2) | 78 (8.3) | 96 (9.2) | 105 (11.9) | 184 (13.9) |  |
| Paternal ACEs | |  |  |  |  |  | <0.001 |
|  | 0 | 57 (13.4) | 80 (8.5) | 53 (5.1) | 34 (3.9) | 53 (4.0) |  |
|  | 1 | 91 (21.4) | 198 (21.1) | 210 (20.1) | 139 (15.8) | 180 (13.6) |  |
|  | 2 | 94 (22.1) | 242 (25.8) | 266 (25.4) | 219 (24.9) | 268 (20.3) |  |
|  | 3 | 82 (19.3) | 184 (19.6) | 222 (21.2) | 178 (20.2) | 280 (21.2) |  |
|  | 4 or more | 101 (23.8) | 235 (25.0) | 295 (28.2) | 311 (35.3) | 541 (40.9) |  |
| Maternal age at the first marriage, years | | 22.0 (20.0-24.0) | 22.0 (20.0-24.0) | 22.0 (20.0-24.0) | 21.0 (20.0-23.0) | 21.0 (19.0-23.0) | <0.001 |
| Paternal age at the first marriage, years | | 23.0 (21.0-25.0) | 23.0 (22.0-25.0) | 23.0 (22.0-25.0) | 23.0 (21.0-25.0) | 23.0 (21.0-25.0) | 0.602 |
| Maternal marriage times | |  |  |  |  |  | 0.338 |
|  | 1 | 415 (97.7) | 912 (97.1) | 1012 (96.8) | 844 (95.8) | 1272 (96.2) |  |
|  | 1 or more | 10 (2.4) | 27 (2.9) | 34 (3.3) | 37 (4.2) | 50 (3.8) |  |
| Paternal marriage times | |  |  |  |  |  | 0.936 |
|  | 1 | 410 (96.5) | 906 (96.5) | 1015 (97.0) | 849 (96.4) | 1278 (96.7) |  |
|  | 1 or more | 15 (3.5) | 33 (3.5) | 31 (3.0) | 32 (3.6) | 44 (3.3) |  |
| Parity | | | |  |  |  | 0.275 |
|  | 0 | 21 (4.9) | 35 (3.7) | 45 (4.3) | 43 (4.9) | 72 (5.5) |  |
|  | 1 | 79 (18.6) | 180 (19.2) | 213 (20.4) | 168 (19.1) | 233 (17.6) |  |
|  | 2 | 186 (43.8) | 380 (40.5) | 395 (37.8) | 334 (37.9) | 499 (37.8) |  |
|  | 3 or more | 139 (32.7) | 344 (36.6) | 393 (37.6) | 336 (38.1) | 518 (39.2) |  |
| History of spontaneous abortion | |  |  |  |  |  | 0.467 |
|  | No | 393 (92.5) | 867 (92.3) | 952 (91.0) | 807 (91.6) | 1195 (90.4) |  |
|  | Yes | 32 (7.5) | 72 (7.7) | 94 (9.0) | 74 (8.4) | 127 (9.6) |  |
| History of stillbirth | |  |  |  |  |  | 0.259 |
|  | No | 420 (98.8) | 921 (98.1) | 1020 (97.5) | 856 (97.2) | 1285 (97.2) |  |
|  | Yes | 5 (1.2) | 18 (1.9) | 26 (2.5) | 25 (2.8) | 37 (2.8) |  |

Note: ACEs, adverse childhood experiences. Continuous variables are presented as medians (M) with interquartile ranges (IQRs), and categorical variables are presented as numbers (N) with percent (%).

Table S4. Association between each type of paternal ACEs and history of spontaneous abortion and stillbirth

| **Types of ACEs** | **Spontaneous abortion** | **Stillbirth** |
| --- | --- | --- |
|  | **OR (95% CI)** | |
| ***Intra-familial*** | | |
| Emotional neglect | 1.05 (0.85 to 1.30) | 0.81 (0.54 to 1.21) |
| Family violence | **1.48 (1.18 to 1.85)** | **0.58 (0.35 to 0.96)** |
| Parental separation or divorce | 2.04 (0.59 to 7.11) | 2.30 (0.29 to 18.06) |
| Parental substance abuse | 1.14 (0.92 to 1.42) | 1.26 (0.85 to 1.89) |
| Parents incarcerated | 0.65 (0.08 to 4.98) | - |
| Parental mental illness | 1.07 (0.77 to 1.50) | 0.61 (0.29 to 1.27) |
| Parental disability | 0.99 (0.76 to 1.28) | 0.97 (0.60 to 1.56) |
| Parental death | 1.08 (0.78 to 1.48) | 0.91 (0.51 to 1.60) |
| Sibling death | 1.03 (0.65 to 1.62) | 1.62 (0.85 to 3.11) |
| Physical abuse | 1.10 (0.89 to 1.37) | 1.05 (0.71 to 1.56) |
| Economic adversity | 0.91 (0.73 to 1.13) | 0.98 (0.66 to 1.47) |
| ***Extra-familial*** | | |
| Bullying | **1.31 (1.03 to 1.68)** | 1.24 (0.78 to 1.98) |
| Loneliness | 0.89 (0.63 to 1.27) | 1.32 (0.77 to 2.27) |
| Community violence | 1.11 (0.79 to 1.57) | 0.47 (0.20 to 1.09) |

Note: ACEs, adverse childhood experiences. OR, odds ratio. CI, confidence interval. All OR values were adjusted for maternal ACEs, maternal age, maternal residence, maternal education, maternal age at the first marriage, age at menarche, maternal health status in childhood, parity, and maternal marriage times, as well as paternal age, paternal education, paternal age at the first marriage, paternal health status in childhood, and paternal marriage times. Bolded means statistically significant. “-” refers to insufficient statistical power.


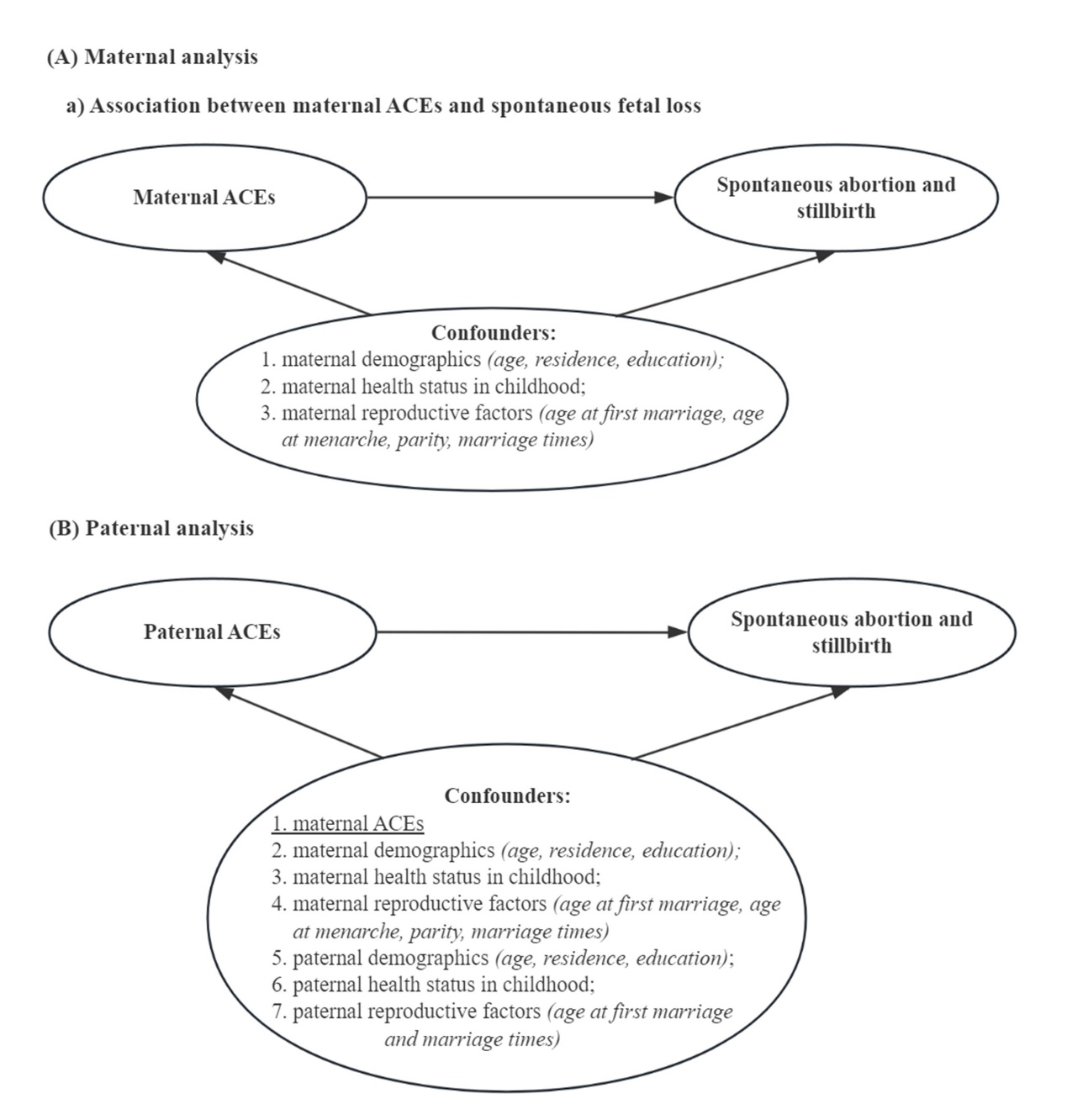


Figure S1. Directed Acyclic Graph of the study hypothesis

Note: ACEs, adverse childhood experiences.
